# Pitfalls of Breast Evaluation in the Emergency Department

**DOI:** 10.7759/cureus.10612

**Published:** 2020-09-23

**Authors:** James T Roberts, Wendy Digiacinto, Quan D Nguyen

**Affiliations:** 1 Diagnostic Radiology, University of Texas Medical Branch, Galveston, USA; 2 Emergency Medicine, University of Texas Medical Branch, Galveston, USA; 3 Radiology, University of Texas Medical Branch, Galveston, USA

**Keywords:** breast abscess, breast cancer, invasive ductal cell carcinoma, emergency department, radiology, breast disease, breast mass, mammogram, ultrasound, breast screening

## Abstract

Breast complaints in the emergency department (ED) include trauma, infection, pain, masses, and nipple discharge. Breast cancer mimics other inflammatory conditions such as mastitis and abscess. Differentiating infectious processes versus cancer can become problematic when no imaging is used. While mammogram is included in the initial imaging for outpatient breast evaluation, ultrasound is more available in the ED. We present a case of a patient seen in the ED for breast pain and mass. The patient had no imaging done, yet the ED physician attempted to drain the mass unsuccessfully and prescribed antibiotics. The patient followed up at a breast center where clinical Stage IIA right breast invasive ductal carcinoma (IDC) was diagnosed. This case highlights the importance of breast imaging before drainage for suspected abscess and the importance of follow-up for all breast complaints that present in the ED to rule out a malignancy.

## Introduction

Breast abscesses can develop from mastitis, cellulitis, or as a primary breast infection. Clinically an abscess will appear as painful inflammation, localized to one area of the breast and may present with induration, drainage, fluctuance, erythema, or fever. Risk factors include smoking, obesity, and African American race. Treatment includes antibiotics and surgical drainage by needle aspiration or surgically [1,2]. Ultrasound is a readily available imaging modality in the emergency department (ED) for breast complaints. Three retrospective studies evaluated the use of ultrasound for suspected breast abscess in the ED. Porembka et al. suggested patient selection for emergent ultrasound can be improved for suspected abscess through risk stratifying patients by factors such as smoking and diabetes, and physical exam findings of induration, fluctuance, erythema, and drainage [3]. Moseley et al. found after hour breast ultrasound use had clinical impact with ultrasound confirmed abscesses more likely to have intervention than those without imaging [4]. Bosma et al. highlighted the need for close follow-up for breast complaints in the ED as breast cancer can mimic infectious processes and suggested additional clinician and patient education for these complaints [5]. The patient in the following case presented with breast pain and mass but no skin symptoms. With no risk factors, the patient could have been managed outpatient at a breast center without attempt at drainage and antibiotics for suspected abscess. The following case demonstrates the need for breast imaging before intervention for suspected abscess and the importance of outpatient follow-up for all breast complaints in the ED.

## Case presentation

The patient is a 34-year-old female who presented to the ED with a one-day history of severe right breast pain with a mass. The patient noted a pea sized mass four months ago. Attributing the mass to her menstrual cycle, she did not seek medical attention until this ED encounter. The patient is not currently on birth control but has taken oral contraceptives in the past. She has never been pregnant and is a nonsmoker with no family history of breast or ovarian cancer. She has no history of breast surgery and menarche was at 13 years old. On exam, there was a palpable, dominant mass in the right breast at the 12 o'clock position measuring 4 cm by 2.5 cm in greatest dimension. The mass was mobile and there was no induration, drainage, fluctuance, erythema skin dimpling, nipple retraction, nipple discharge, peau d'orange, palpable lymphadenopathy in the bilateral axilla(s), lymphedema of the bilateral arm, or palpable masses in the left breast. The ED physician attempted to aspirate and drain the mass, which was unsuccessful. She was then given Motrin and clindamycin for suspected abscess and told to follow up at a breast center for imaging. No imaging was done in the ED. Three weeks later the patient was seen at a breast health and imaging center and completed a mammogram, ultrasound, and biopsy.

**Figure 1:**
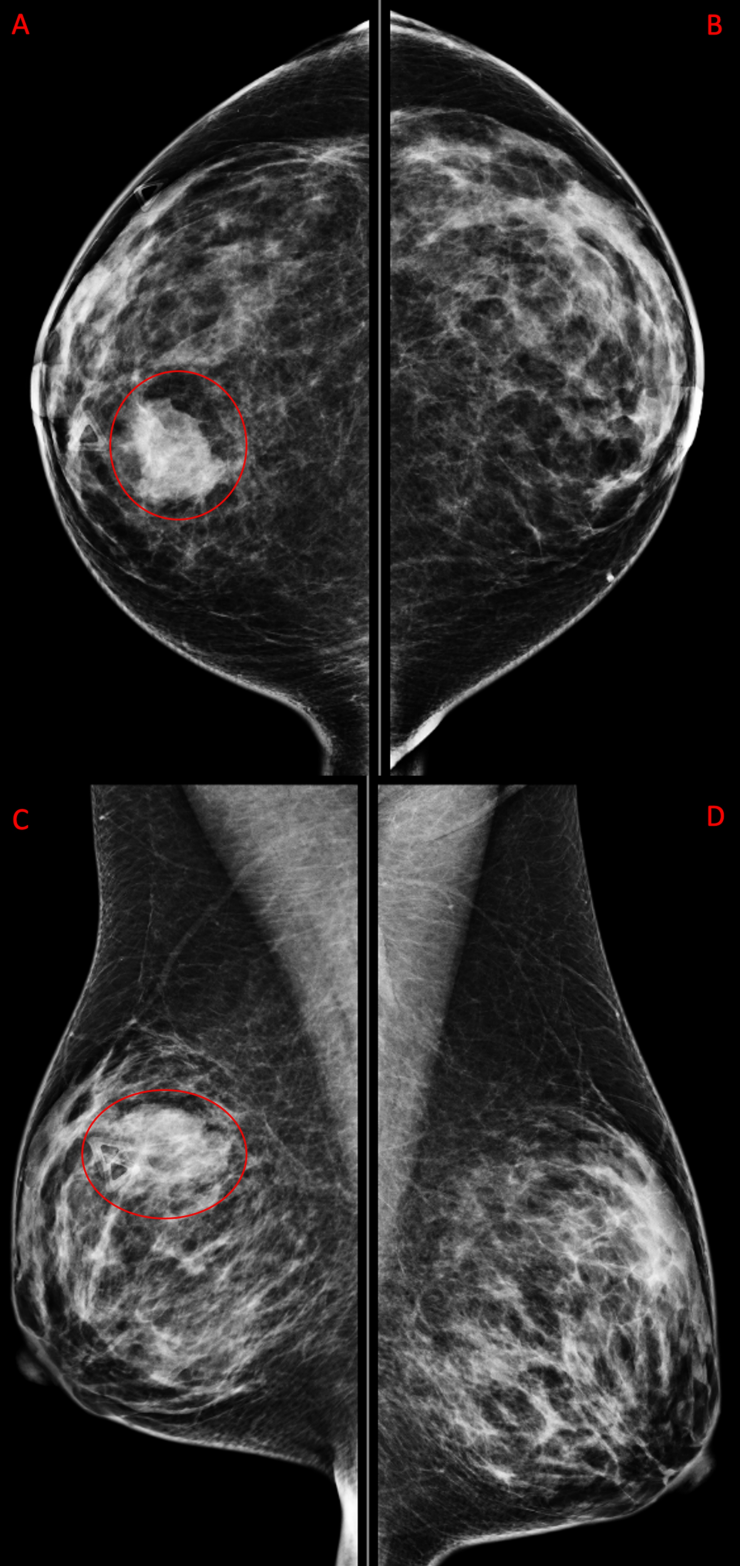
Diagnostic Mammogram Diagnostic mammogram with triangle marker at the site of palpable mass. Right breast has irregular mass with microlobulated margins measuring 29 mm (red circles) seen in craniocaudal (A) and mediolateral oblique (C) views. The left breast includes craniocaudal (B) and mediolateral oblique (D) views. Left breast is negative for any pathology.

**Figure 2:**
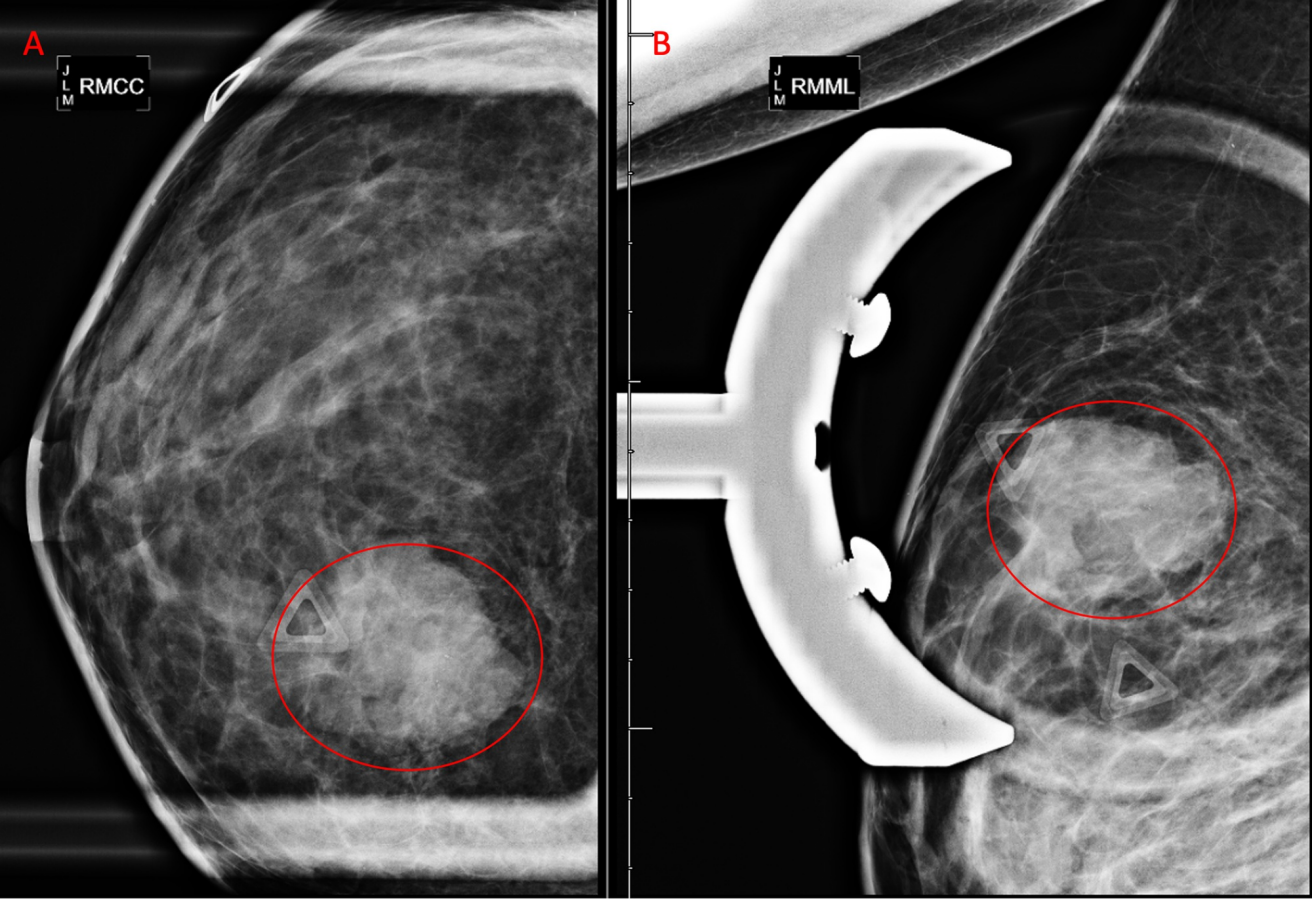
Diagnostic Mammogram Right Breast Spot Compression Views with Triangle Marker at the Site of Palpable Mass Right breast irregular mass with microlobulated margins measuring 29 mm (red circles) shown in craniocaudal (A) and mediolateral oblique (B) views.

Mammogram demonstrated an irregular mass measuring 29 mm in longest dimension at 12 o'clock at a distance of 7 cm from the nipple at the site of palpable mass (Figure 1 and Figure 2). Coarse heterogeneous calcifications were noted in the mass. The breast was heterogeneously dense which may have lowered the sensitivity of mammography.

**Figure 3:**
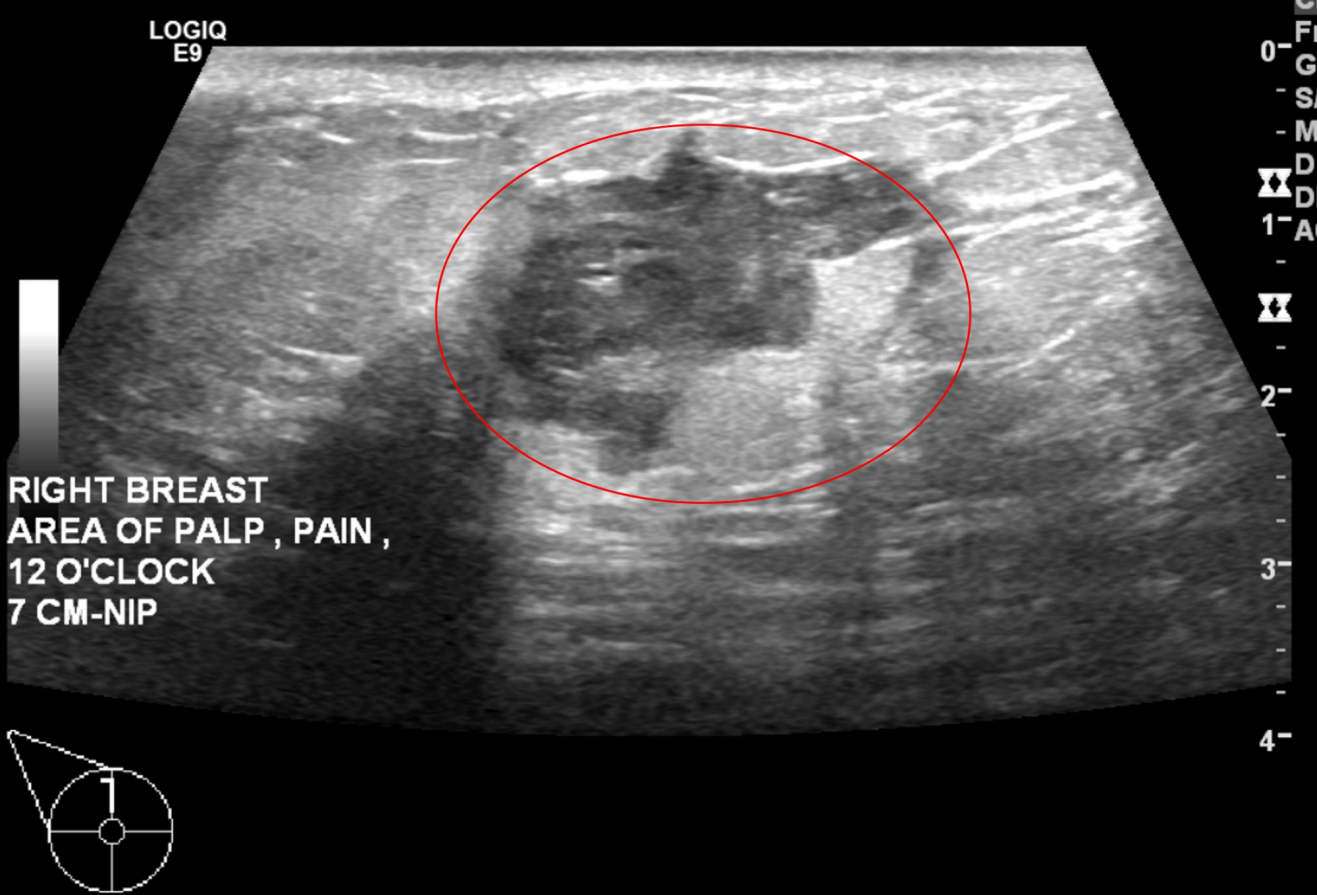
Diagnostic Ultrasound of Right Breast Right breast irregular mass with angular and microlobulated margins measuring 29 x 17 x 28 mm (red circle).

**Figure 4:**
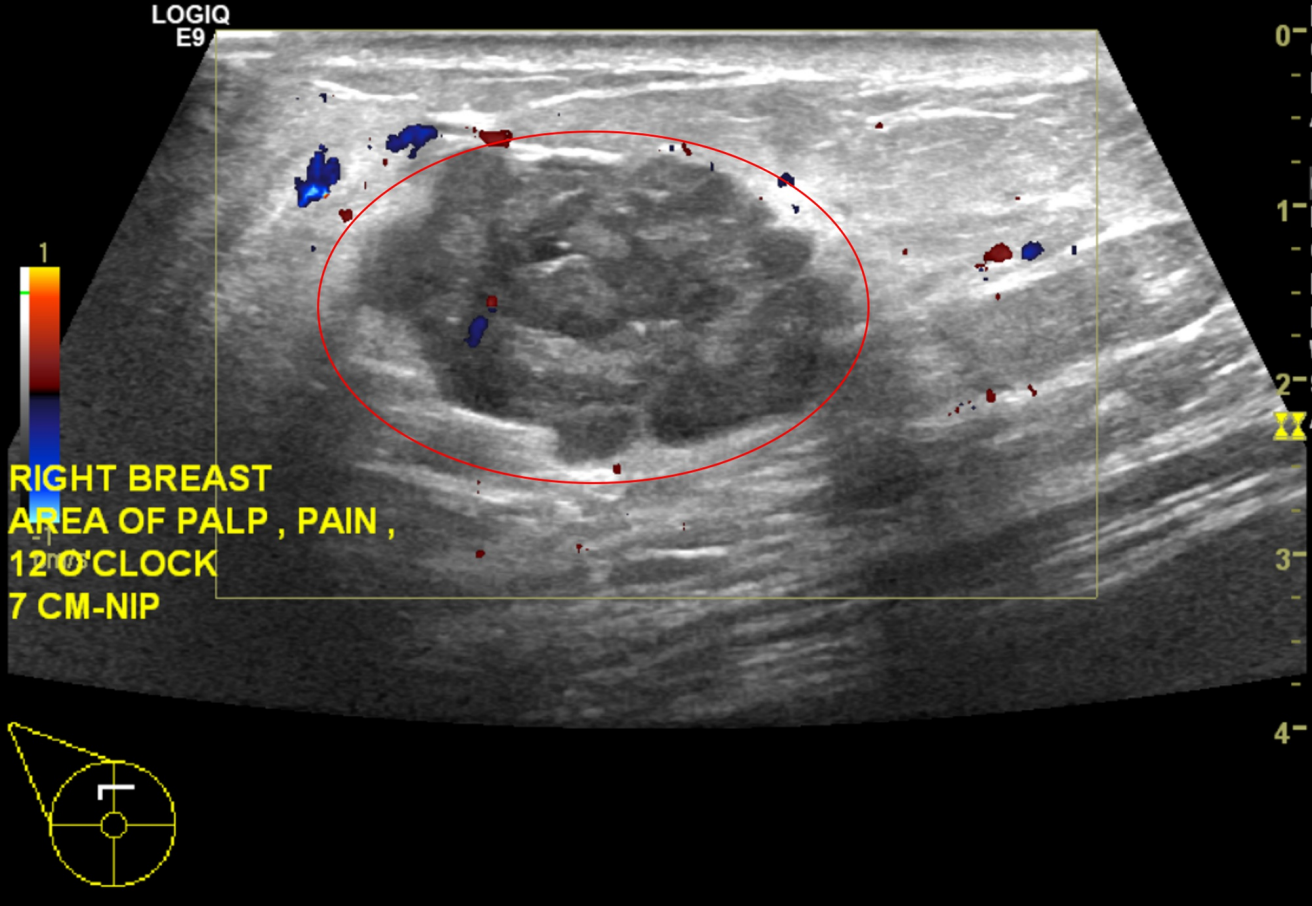
Right Breast Diagnostic Ultrasound with Color Doppler in the Transverse View Right breast irregular mass with angular and microlobulated margins measuring 29 x 17 x 28 mm (red circle).

**Figure 5:**
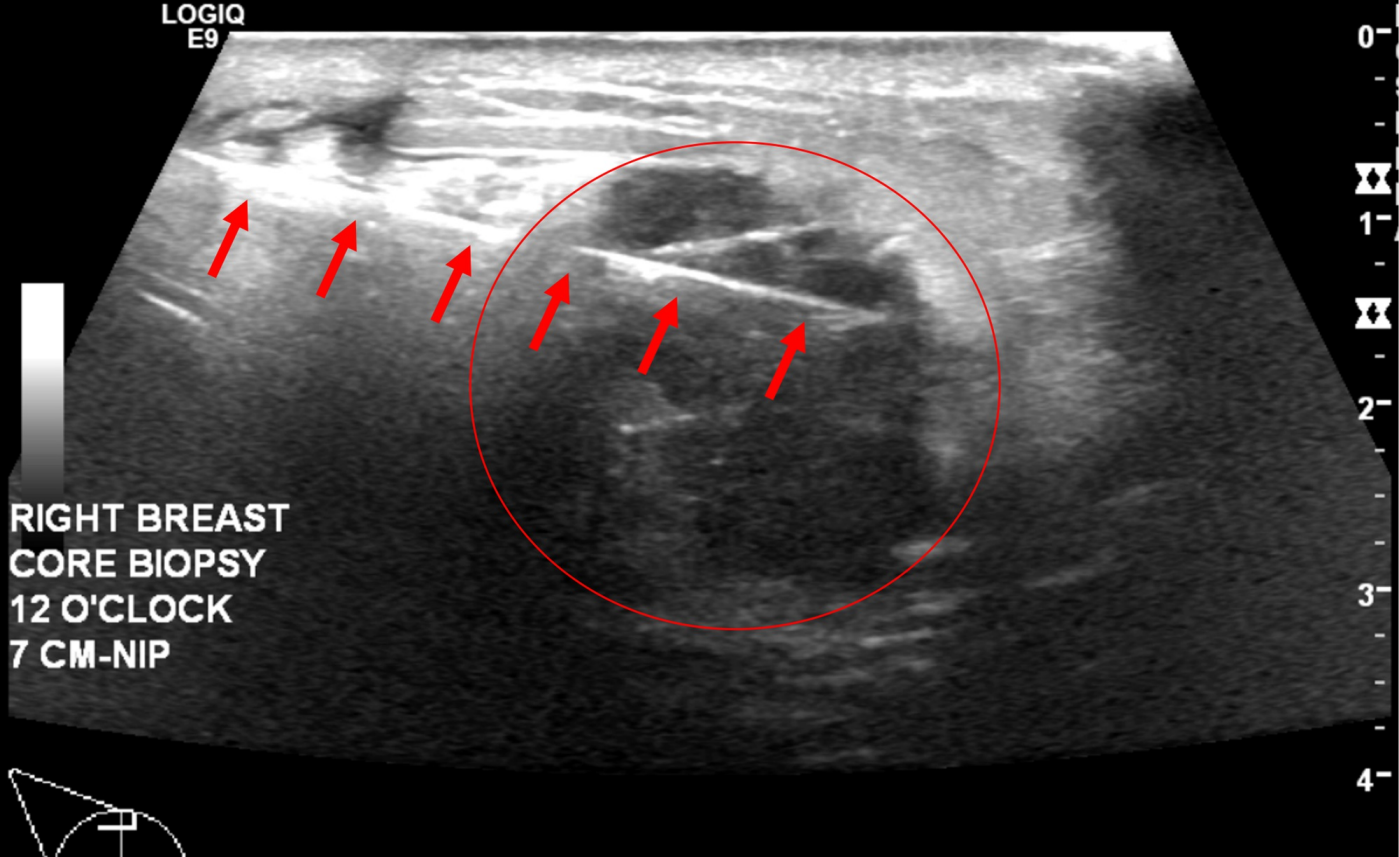
Ultrasound Guided Core Biopsy, Transverse View Hyperechoic needle (red arrows) through the right breast mass (red circle).

Right breast ultrasound demonstrated a solid mass measuring 29 x 17 x 28 mm at the site of the clinically palpable mass at 12 o'clock at a distance of 7 cm from the nipple (Figure 3 and Figure 4). The characteristics of the finding included angular margins, an orientation that is not parallel to the skin line and an irregular shape. Findings were highly suggestive of malignancy, Breast Imaging-Reporting and Data System (BIRADS) Category 5. Ultrasound-guided core biopsy of the right breast mass was recommended (Figure 5). Ultrasound of the right axilla was unremarkable.

Clinical stage IIA right breast invasive ductal carcinoma was diagnosed with biopsy.

## Discussion

An abscess is a soft tissue infection described as a localized collection of pus within the dermis or subcutaneous space commonly due to staphylococcus aureus, both methicillin susceptible and resistant [6]. Breast abscesses can be a sequela of mastitis, cystic disease, neoplasm, trauma, or surgery. Clinically it will appear as a fluctuant, warm, erythematous mass that is painful. Sonography can be used to accentuate size and location and will show an “indistinct mass with centric anechoic or hypoechoic regions with septae and/or low-level internal echoes with or without debris levels” [2]. Few practices maintain a breast-certified technologist or breast fellowship trained radiologist in the ED. Consequently, the level of sonographer and radiologist training and experience will vary, predominantly on nights and weekends.

The purpose of this case is to caution emergency room physicians that palpable lumps may be misdiagnosed as abscess when there is actually an underlying breast cancer. It is ideal to have breast symptoms evaluated by a breast sonographer and breast faculty, but these services are typically only available during outpatient clinic hours. Initial workup for suspected breast abscess with drainage and antibiotics may occur after hours with Emergency Room physicians, but patients should complete an official breast workup with a breast imaging clinic in the outpatient setting.

A retrospective single center study assessed the use of breast sonography in the emergency department and found out of 581 breast ultrasounds for suspected breast abscess in the ED, 74% were negative for abscess, repeat imaging was recommended in 40%, and 21% (6/29) of patients with cancer were read falsely as negative. Ultrasounds were interpreted by breast fellowship-trained radiologists during the weekday and non-breast fellowship-trained radiologists during the night and weekends. Breast fellowship-trained radiologists suggested repeat and/or follow-up imaging on 66% of patients and concluded 5% of the exams technically inadequate compared to 30% and 0% respectively by non-breast fellowship trained radiologists. Of the malignancies, breast imagers missed four of the 21 they interpreted compared to two of the eight by non-breast imagers. The authors suggest that in the same way Ottawa ankle rules stratify those who need ankle imaging, emergent breast ultrasound in the ED should be stratified by risk factors such as smoking and diabetes, and by physical exam findings of induration, fluctuance, erythema, drainage. This study found that if ultrasounds are ordered by this stratification criteria, only 3% of patients with abscesses would not be diagnosed in the ED. Those that do not meet this criterion, such as those that come with pain as the only symptom, would be better managed outpatient at a breast imaging center. However, due to the 21% missed malignancy in this study, they recommend all patients with breast ultrasound completed in the ED follow-up outpatient at a dedicated breast imaging center within 2-4 weeks [3].

In another retrospective study Moseley et al. sought to determine clinical findings suggestive of breast abscess, the value of after-hour use of emergent or urgent breast ultrasonography, and its impact on clinical management. The study showed 26% (n = 100) of the patients who underwent ultrasound had an abscess with risk factors being palpable abnormality and history of breast surgery within eight weeks prior. After hours breast ultrasound had clinical impact with ultrasound confirmed abscesses more likely to have intervention after-hours than those without imaging. The authors stated the study supported after-hour breast ultrasound due to its impact on clinical management [4]. Bosma et al. found 44% (86/185) of patients who underwent ultrasound for suspected abscess in the ED were found to have an abscess, while the remaining were diagnosed with mastitis. Follow-up recommendations were reported verbally to the clinician and documented in the report. One-fourth of cases in this study did not follow up, for which the authors suggested additional clinician and patient education as inflammatory carcinoma can present similar to mastitis and breast abscess. Assessment of complete resolution of symptoms is crucial [5].

While the patient in our case had no delay in follow-up at a breast imaging center, she unnecessarily and unsuccessfully underwent drainage of a suspected abscess that was later proven to be stage IIA right breast invasive ductal carcinoma. She was also unnecessarily started on antibiotics. Had she presented with exam findings of an abscess such as induration, fluctuance, erythema, or drainage an ultrasound would have been appropriate. Since her complaints were pain and mass with no skin involvement, this patient would be better managed at a breast center outpatient for a full workup.

## Conclusions

Excessive utilization, suboptimal studies, and misdiagnosis due to lack of radiology breast expert and breast certified ultrasound technologist after hours and on weekends are risks associated with ultrasonography use in the ED. Risk stratifying breast ultrasound use could alleviate these risks and aid in reduction of misdiagnosis in the ED. Follow-up is extremely important for breast complaints presenting in the ED to rule out malignancy.
